# Topiramate plus citalopram in the treatment of Compulsive-Impulsive Sexual Behaviors

**DOI:** 10.1186/1745-0179-2-9

**Published:** 2006-05-22

**Authors:** Donatella Marazziti, Bernardo Dell'Osso

**Affiliations:** 1 Dipartimento di Psichiatria, Neurobiologia, Farmacologia e Biotecnologie, University of Pisa, Via Roma, 67, 56100 Pisa, Italy; 2 Department of Psychiatry, Compulsive, Impulsive and Anxiety Disorders Program, Mount Sinai School of Medicine, One Gustave L. Levy Place, 10029, New York, NY, USA

## Abstract

Compulsive-Impulsive Sexual Behaviors (C-ISBs) include repetitive sexual acts and compulsive sexual thoughts which occur so frequently and with such intensity that they interfere with sexual intimacy and interpersonal and occupational functioning and whose categorization and effective treatments are still unclear. We report the case of a patient affected by C-ISBs and bipolar disorder of type II who improved dramatically after three months' addition of topiramate to citalopram. Topiramate is a powerful anticonvulsant which has recently been proposed also for the treatment of migraine, bipolar disorder and binge eating disorder. This case-report suggests that topiramate might be beneficial in augmentation with citalopram in patients suffering from C-ISBs, although controlled studies to confirm our findings are needed.

## Intrroduction

Compulsive-Impulsive Sexual Behaviors (C-ISBs) include repetitive sexual acts and compulsive sexual thoughts. The individual feels compelled or driven to perform the behavior, which may or may not cause subjective distress. Although generally not ego-dystonic, the behavior may interfere with several aspects of the patient's life, causing social or occupational impairment, or legal and financial consequences [1,2]. C-ISBs involve a broad range of paraphilic or nonparaphilic symptoms [3]. Paraphilic C-ISBs involve unconventional sexual behaviors in which there is a disturbance in the object of sexual gratification or in the expression of sexual gratification (e.g., exhibitionism, voyeurism). Nonparaphilic C-ISBs, on the other hand, involve conventional sexual behaviors that have become excessive or uncontrolled [3]. The true prevalence of C-ISBs remains unknown, given the hetereogeneity of these disorders as well as the secretiveness of the condition for the majority of the afflicted patients. Investigations conducted in the early 1990s reported prevalence estimates of I-CSBs ranging from 5% to 6% of the US population [3,4]

Although C-ISBs seem relatively common, controlled trials on pharmacological treatments for these disorders are still lacking, and the available literature on this topic consists essentially of open-label and case-report series. Positive findings have been reported with lithium and tricyclics [5-7], selective serotonin reuptake inhibitors [8-11], buspirone [12,13] and nefazodone [14]. In addition, the opioid antagonist naltrexone has recently been shown to be efficacious in some case-reports [15]. Finally, different forms of psychotherapy have been shown to be effective for specific subtypes of C-I sexual behaviors [16].

Topiramate is an orally active anticonvulsant whose mechanisms of action include potentiation of GABA to activate GABA_A _receptors and antagonism of glutamate at specific receptors [17,18]. Although the efficacy of topiramate has not been evaluated yet in the treatment of C-ISBs, previous reports suggest that this compound might be beneficial in a variety of psychiatric illnesses including affective disorders [19,20], eating disorders [21], post-traumatic stress disorder [22] and obsessive-compulsive disorder (OCD) [23]. In addition, some authors [24] have reported an overlap between C-ISBs and OCD and included C-ISBs in a broader obsessive-compulsive spectrum including also impulse-control disorders such as pathological gambling (PG), eating disorders such as binge eating disorder (BED), and other mental conditions such as body dysmorphic disorder and autism. Finally topiramate' putative mood stabilizer properties [19,20] may represent an additional advantage for its use in subjects with comorbid bipolar disorder, like the patient we report here who was successfully treated with a pharmacological augmentation of topiramate plus citalopram.

### Case report

Mr. A, male, 32 years old, had been suffering since his late teens from C-ISBs in the form of episodes (5–7 a year lasting from 7 to 10 days) of hypersexuality characterized by repetitive masturbation and sexual intercourse (up to 25–30 times a day). On reaching his twenties, having finished school and with a job and salary, he started to meet prostitutes. Episodes became more frequent and his need of compulsive sexual intercourse (up to 30 times in one day) was so intense that he could waste all his salary in just a few days. He developed a depressive episode requiring hospitalization in a day-hospital. The patient was diagnosed as suffering from C-ISBs and bipolar disorder of type II and was initially treated with sertraline (100 mg/d) and lithium salts (900 mg/d). After two months, the depressive symptoms had disappeared, but the patient refused to continue with the mood stabilizer because of significant weight gain (8 kilos). During the subsequent years he presented episodes of opposed polarity, with the presence throughout of C-ISBs, egodystonic when depressed and egosyntonic when euphoric, but he could not take any of the prescribed mood stabilizers, including valproate, carbamazepine, oxcarbamazepine, lamotrigine, or gabapentin, because of various side effects.

His most recent severe depressive episode occurred in the autumn of 2002: he had been depressed after spending his entire salary in just one day with prostitutes, and, on that occasion, had expressed suicidal intention. He was treated with benzodiazepines (only in the first week) and citalopram 20 mg/d, titrated up to 60 mg/d in two weeks. Then adjunctive topiramate was added to his current treatment at an initial dosage of 50 mg, which was titrated up to 200 mg/d in one month. After three months of this pharmacological regimen, depressive symptoms and sexual urges were significantly decreased and citalopram was reduced progressively until cessation. The patient was maintained on topiramate only which, given the mild mood shifts, was increased to 400 mg/d and continued for one year, with no relevant side-effects. During this period the patient experienced for the first time in his life a long period of stability with a normal arousal pattern, and he began a loving relationship which is still ongoing.

## Discussion

C-ISBs are characterized by abnormal sexual behaviour in terms of frequency and intensity of arousal patterns, which provokes significant subjective sufferance, as well as impairment of social relationship and work activities. In addition, the stigma related to sexual (and mental) disturbances understandably represents an obstacle to reliable information on the prevalence of C-ISBs and this also provides an explanation of the few clinical studies in the field.

Although prevalent and impairing, C-ISBs have not been systematically investigated form a pharmacological point of view, and different medications have demonstrated to be effective, although generally not in randomised controlled trials [25]. The efficacy of many pharmacological treatments in C-ISBs could also reflect the various dimensional approaches to C-ISBs in terms of psychopathologic and clinical similarities and overlaps with other Axis I disorders. In fact, the most consistent findings on this topic tend to categorize C-ISBs either as an impulse control disorder or as an entity belonging to the bipolar spectrum disorders: this represents the rationale for the use of mood stabilizers in C-ISBs.

The case report described herein underlines this link between C-ISBs and bipolar spectrum disorders and suggests that, amongst mood stabilizers, topiramate might be particularly useful in the broader area of impulse control disorders. However, topiramate-induced cases of hypomanic and manic episodes have also been reported in literature [26,27]. Nevertheless, the pharmacological profile of topiramate including a rapid onset of action along with a low weight gain liability and sexual dysfunction may represent a further interesting feature for future research with this drug.

## References

[B1] Black DW Kehrberg LLD Flumerfelt DL Characteristics of 36 subjects reporting compulsive sexual behavior Am J Psychiatry 1997 154 243 249 9016275 10.1176/ajp.154.2.243

[B2] Black DW Compulsive sexual behavior: a review J Pract Psychiatry Behav Health 1998 4 219 229

[B3] Coleman E Compulsive Sexual Behavior. New Concepts and Treatments J Psychol Hum Sexual 1991 4 37 52 10.1300/J056v04n02_04

[B4] Schaffer SD Zimmerman ML The sexual addict: a challenge for the primary care provider Nurse Pract 1990 15 25 33 2356035

[B5] Cesnik JA Coleman E Use of lithium carbonate in the treatment of autoerotic asphyxia Am J Psychoter 1989 43 277 285 10.1176/appi.psychotherapy.1989.43.2.277 2502035

[B6] Coleman E Cesnik J Moore A An exploratory study of the role of psychotropic medications in treatment of sex offenders J Offend Rehabil 1992 18 75 88 10.1300/J076v18n03_08

[B7] Kruesi MJ Fine S Valladres L Paraphilias: A double-blind cross-over comparison of clomipramine versus desipramine Arch Sex Behav 1992 21 587 593 10.1007/BF01542257 1482282

[B8] Emmanuel NP Lydiard RB Ballenger JC Fluoxetine treatment of voyerism Am J Psychiatry 1991 148 950 2053642 10.1176/ajp.148.7.950a

[B9] Kafka MP Sertraline pharmacotherapy for paraphilias and paraphilia-related disorders: an open trial Ann Clin Psychiatry 1994 6 189 195 7881500 10.3109/10401239409149003

[B10] Stein DJ Hollander E Anthony DT Serotonergic medications for sexual obsessions, sexual addiction, and paraphilias J Clin Psychiatry 1992 53 267 271 1386848

[B11] Fedoroff JP Serotonergic drugs treatment of deviant sexual interests Annals Sex Res 1993 6 105 121 10.1007/BF00849302

[B12] Fedoroff JP Buspirone hydrochloride in the treatment of transvestic feticism J Clin Psychiatry 1988 49 408 409 3271001

[B13] Fedoroff JP Buspirone hydrochloride in the treatment of atypical paraphilia Arch Sex Behav 1992 21 401 406 10.1007/BF01542028 1497477

[B14] Coleman E Gratzer T Nesvacil L Nefazodone and the treatment of nonparaphilic compulsive sexual behavior: a retrospective study J Clin Psychiatry 2000 61 282 284 10830149

[B15] Raymond NC Grant JE Kim SM Treatment of compulsive sexual behavior with naltrexone and serotonin reuptake inhibitors: two case studies Int Clin Psychopharmacol 2002 17 201 205 10.1097/00004850-200207000-00008 12131605

[B16] Coleman E Rosen RC SR, Leiblum SR Treatment of compulsive sexual behavior Case Studies in Sex Therapy 1995 New York, NY: Guildford Press 333 349

[B17] White HS Brown SD Woodhead JH Topiramate modulates GABA-evoked currents in murine cortical neurons by non-diazepine mechanism Epilepsia 2000 41 S17 S20 10.1111/j.1528-1157.2000.tb02165.x 10768294

[B18] Gibbs JW Sombati S DeLorenzo RJ Cellular actions of Topiramate: blockade of kainate-evoked inward currents in cultured hippocampal neurons Epilepsia 2002 41 10 S16 10.1111/j.1528-1157.2000.tb02164.x 10768293

[B19] McElroy SL Suppes T Keck PE Open-label adjunctive topiramate on the treatment of bipolar disorders Biol Psychiatry 2000 47 1025 1033 10.1016/S0006-3223(99)00316-9 10862801

[B20] Calabrese JR Keck PE Jr McElroy SL Shelton MD A pilot study of topiramate as monotherapy in the treatment of acute mania J Clin Psychopharmacol 2001 21 340 342 10.1097/00004714-200106000-00015 11386499

[B21] McElroy SL Arnold LM Shapira NA Topiramate in the Treatment of Binge-Eating Associated with Obesity (poster) 53rd Institute on Psychiatric Services 2001 Orlando

[B22] Berlant J vanKammen DP Open-label Topiramate as Primary or Adjunctive therapy in Chronic Civilian Posttraumatic Stress Disorder: A Preliminary report J Clin Psychiatry 2002 63 15 20 11838620 10.4088/jcp.v63n0104

[B23] Hollander E Dell'Osso B Topiramate plus Paroxetine in treatment-resistant obsessive-compulsive disorder Int Clin Psychopharmacol 2006 21 3 189 191 10.1097/01.yic.0000199453.54799.cc 16528143

[B24] Hollander E Obsessive-Compulsive Related Disorders 1993 Washington DC American Psychiatric Press

[B25] Dell'Osso B Altamura AC Allen A Marazziti D Hollander E Epidemiologic and clinical updates on impulse control disorders: a critical review Eur Arch Psychiatry Clin Neurosci 2006 in press 10.1007/s00406-006-0668-0 PMC1705499 16960655

[B26] Jochum T Bar KJ Sauer H Topiramate induced manic episode J Neurol Neurosurg Psychiatry 2002 73 2 208 9 10.1136/jnnp.73.2.208 12122191 PMC1737976

[B27] Kaplan M Hypomania with topiramate J Clin Psychopharmacol 2005 25 2 196 7 10.1097/01.jcp.0000155828.82456.68 15738759

